# Death in a Dental Office

**Published:** 1871-10

**Authors:** 


### EDITORIAL DEPARTMENT.

---

*Death in a Dental Office.*—Miss Julia W. Doolittle, aged 31 years, of Hudson, near Fulton Avenue, Brooklyn, N. Y., called on Dr. Brewster, Dentist, on the 27th inst, to have a number of teeth extracted, preparatory to having made a set of artificial teeth. She had called on Dr. Brewster the Saturday previous, and said that she would be unable to submit to the operation without taking nitrous oxide gas or chloroform. He finally administered gas, but it did not have the desired effect. She wanted to take chloroform then, but Dr. B. refused to administer it, and informed her that if she persisted in taking it she would have to bring a physician with her who would assume the responsibility. An engagement was accordingly made for the morning of the 25th, at 9½ o'clock, and at that time Miss Doolittle called at the office and met Dr. S. G. Little, of Carlton Avenue, one of the most experienced physicians in the city. The doctor asked Miss Doolittle whether she was perfectly healthy, and she said that she was, but that she had slight palpitation of the heart, which she did not consider as anything serious, Dr. Little then administered four drachms of chloroform to her in the presence of Dr. Brewster and his assistant, who were in readiness to extract the teeth. After the teeth were drawn, the physician suddenly announced that the woman's pulse was becoming weak, and that she was sinking rapidly. They became alarmed and began to apply restoratives, but without effect. Several other medical men were summoned, and a galvanic current was used for more than an hour, but all efforts to revive the woman failed and she died. Dr. Little was shocked at the fatal result of the operation. He said that Miss Doolittle had called at his office on the Saturday previous, and made an appointment with him to administer chloroform to her at Dr. Brewster's. He met her there and administered four drachms of chloroform on a napkin. When she was under the influence of the drug the dentist began to extract her teeth. The doctor finding the pulse growing weak began to apply restoratives, but without effect. He had known Miss Doolittle about a month or six weeks only. She was a native of Connecticut, and had lived in Brooklyn ten or twelve years, employed in a sewing machine establishment, at a good salary, where she was considered a first class operator. The body was removed to the house of a relative, where a coroner will empannel a jury preparatory to an inquest.

